# Survival of enterohemorrhagic *Escherichia coli* in the presence of *Acanthamoeba castellanii* and its dependence on Pho regulon

**DOI:** 10.1002/mbo3.40

**Published:** 2012-10-30

**Authors:** Samuel Mohammed Chekabab, France Daigle, Steve J Charette, Charles M Dozois, Josée Harel

**Affiliations:** 1 Centre de Recherche en Infectiologie Porcine (CRIP), Faculté de Médecine Vétérinaire, Université de MontréalSaint-Hyacinthe, Québec, Canada; 2 Department of Microbiology and Immunology, Université de MontréalMontréal, Québec, Canada; 3 Institut de Biologie Intégrative et des Systèmes, Pavillon Charles-Eugène-Marchand, Université LavalQuébec City, Québec, Canada; 4 Centre de recherche de l'Institut universitaire de cardiologie et de pneumologie de Québec (Hôpital Laval)Québec City, Québec, Canada; 5 Départment de biochimie, de microbiologie et de bio-informatique, Faculté des sciences et de génie, Université LavalQuébec City, Québec, Canada; 6 INRS-Institut Armand-FrappierLaval, Québec, Canada

**Keywords:** *Acanthamoeba castellanii*, bacterial persistence, enterohemorrhagic *Escherichia coli*, Pho regulon, Shiga toxin

## Abstract

Enterohemorrhagic *Escherichia coli* (EHEC) are involved in outbreaks of food-borne illness and transmitted to humans through bovine products or water contaminated by cattle feces. Microbial interaction is one of the strategies used by pathogenic bacteria to survive in the environment. Among protozoa, the free-living amoebae are known to host and protect several water-borne pathogens. In this study, the interaction between EHEC and the predacious protozoa *Acanthamoeba castellanii* was investigated. Using monoculture and cocultures, growth of both organisms was estimated for 3 weeks by total and viable cell counts. The numbers of EHEC were significantly higher when cultured with amoebae than without, and less EHEC shifted into a viable but nonculturable state in the presence of amoebae. Using several mutants, we observed that the Pho regulon is required for EHEC growth when cocultured with amoebae. In contrast, the Shiga toxins (Stx) were not involved in this association phenotype. Cocultures monitored by electron microscopy revealed a loss of the regular rod shape of EHEC and the secretion of multilamellar vesicles by the amoebae, which did not contain bacteria. As the interaction between *A. castellanii* and EHEC appears beneficial for bacterial growth, this supports a potential role for protozoa in promoting the persistence of EHEC in the environment.

## Introduction

Enterohemorrhagic *Escherichia coli* (EHEC) are a group among Shiga toxin (Stx)-producing *E. coli* involved in outbreaks of food-borne illness. It is assumed that Stx2 plays a pivotal role in the development of hemolytic uremic syndrome (HUS), a possible life-threatening complication that can arise from EHEC infection. EHEC cause attaching/effacing of microvilli on enterocytes in humans, resulting in acute watery diarrhea. This is linked to the *locus of enterocyte effacement*, which encodes the Eae adhesin and type III secretion system and some of its effectors (Gyles 2007; Melton-Celsa et al. 2012).

Enterohemorrhagic *Escherichia coli* are widely distributed in domestic ruminants that are considered an important route for transmission to humans (Hancock et al. 2001). Cattle are the main reservoir for EHEC, especially serotype O157:H7, and can result in zoonotic transmission through the consumption of raw or undercooked contaminated beef and other bovine products. However, fresh salad greens, fruit juices, unpasteurized milk, and water contaminated by cattle manure are other sources of human infection by EHEC. The persistence of this dangerous human pathogen in animals, animal products, plants, soil, and water is well established (Maule 2000; Islam et al. 2004).

Free-living protozoa are also found in a diverse range of habitats, from water and soils to the intestinal tract of a range of vertebrate hosts (Barker and Brown 1994). They participate in nutrient and energy turnover acting as predators controlling bacterial numbers, but they also sometimes serve as reservoirs for pathogenic microorganisms. Thus, protozoa could contribute to transmission of the bacteria. In fact, certain bacteria have been shown to survive ingestion by protozoa (Huws et al. 2008). Bacterial pathogens including *Vibrio*, *Legionella*, *Mycobacterium, enteropathogenic E. coli,* and the meningitis-causing *E. coli* strain K1 multiply and/or survive within protozoa (King et al. 1988; Barker and Brown 1994; Fields 1996; Steinert et al. 1998; Abd et al. 2005; Alsam et al. 2006).

The *Acanthamoeba* species, which are free-living amoebae, are naturally occurring hosts of several water-borne pathogens (Sandstrom et al. 2010; Cateau et al. 2011). These protozoa are known to resist various stress conditions (Coulon et al. 2010; Mogoa et al. 2010, 2011). They are commonly found in water and in other habitats and may therefore coexist in environments with pathogens such as *E. coli*. Consequently, it has been shown that increased survival of *E. coli* O157 occurs in association with the common environmental protozoan *Acanthamoeba polyphaga* (Barker et al. 1999). Moreover, transcriptomic investigation showed that expression of Stx by *E. coli* O157 was increased during short-term interaction with *Acanthamoeba castellanii* (Carruthers et al. 2010). However, the study on long-term interaction on weeks of *Acanthamoeba* with *E. coli* O157 has never been addressed before.

Phosphate, an essential component of bacterial nutrition, plays an important role in cell metabolism. It is a constituent of nucleic acids, phospholipids, and lipopolysaccharides and is involved in energy transport and many catalytic processes. Bacteria meet their requirements for phosphorus through the assimilation of various phosphorus-containing compounds. Such compounds are transported into cells and then incorporated into ATP (Wanner 1993). The preferable source of phosphorus in bacteria is inorganic phosphate (Pi), and under phosphate-limiting conditions, Pi is transported by the phosphate-specific transport (Pst) system while the phosphate regulon (Pho) allows bacteria to adapt to low Pi concentration but also is shown to modulate bacterial virulence (Crepin et al. 2011).

Our hypothesis is that *Acanthamoeba* might be a reservoir of EHEC especially in aquatic environments. Moreover, in these environments, limited nutrient availability such as low Pi could be a nutritive stress for microorganisms. This study was designed to characterize the long-term in vitro association of EHEC strain EDL933 (Riley et al. 1983) with *A. castellanii*. The specific aims were to assess the involvement of the Pho regulon in these interactions and to determine the impact of Stx toxins on EHEC/amoeba interactions.

## Materials and Methods

### Culture of amoebae

In this study, we used the free-living amoebae *A. castellanii* genotype T4 (American Type Culture Collection 50492) cultured as previously described (Siddiqui et al. 2011). Briefly, amoebae were grown without shaking in 30 mL of PYG medium (0.75% [w/v] proteose peptone, 0.75% [w/v] yeast extract, and 1.5% [w/v] glucose) in T-75 tissue culture flasks at 30°C (Douesnard-Malo and Daigle 2011).

### Bacterial strains

All strains used in this study are listed in Table 1. We used the EHEC O157:H7, strain EDL933 isolated from contaminated hamburger during a food outbreak in 1982 and implicated in HUS cases (Riley et al. 1983). *Escherichia coli* HB101, a K-12 laboratory strain, was used as negative control. EDL933Δ*stx* (Δ*stx1,* Δ*stx2* double mutant) was graciously provided by Dr. Christine Martin (INRA).

**Table 1:** *Escherichia coli* strains and plasmid used in this study

Strain or plasmid	Description and relevant characteristics^1^	Source or reference
Enterohemorrhagic *Escherichia coli* strains		
EDL933WT	*E. coli* O157:H7; wild type	ATCC 700927 (Strockbine et al. 1986)
EDL933*Δstx*	EDL933; *Δstx1/Δstx2;* Stx negative	Gobert et al. (2007)
EDL933*Δpst*	EDL933; *pstCAB::Km*; Pho regulon constitutive	This study
EDL933*ΔphoB*	EDL933*; phoB::Km*; Pho regulon negative	This study
*E. coli* laboratory strains		
K-12 HB101	F^−^ *mcrB mrr hsdS20*(r_B_^−^ m_B_^−^) *recA13 leuB6 ara-14 proA2 galK2 xyl-5 mtl-1 rpsL20*(Sm^R^) *glnV44* λ^−^ *lacY*	Laboratory stock
SM10λpir	*thi-1 leu tonA lacY supE recA:: RP4-2-Tc::Mu λpir,* Km^r^	Laboratory stock
χ7213	SM10λpir *ΔasdA4,* Km^r^	Kang et al. (2002)
Plasmid		
pKNG101	Suicide vector, *sacB* Sm^r^	Kaniga et al. (1991)
pKNG800K	*pKNG101, pstCAB:: km sacB,* Sm^r^Km^*r*^	Lamarche et al. (2005)
pKD13	Template plasmid, *Km*^*r*^gene flanked by FRT sites. *Ap*^*r*^*, Tet*^*r*^	*AY048744* (Datsenko and Wanner 2000)
pMEG-375	*sacRB mobRP4 oriR6K* Cm^r^, Ap^r^	S. Tinge, Megan Health Inc

1 Km^r^, kanamycin resistant; Cm^r^, chloramphenicol resistant; Sm^r^, streptomycin resistant; Tet^r^, tetracycline resistant, Ap^r^; ampicillin resistant.

To investigate the role of the Pho regulon, we created the isogenic mutants Δ*pstCAB* (constitutive Pho) and Δ*phoB* (inactivated Pho) in EDL933. The *pst* knockout mutant was obtained as previously described (Lamarche et al. 2005). Briefly, the pKNG800K suicide vector (Miller and Mekalanos 1988) containing the Δ*pstCAB*::km construct was transferred to strain SM10λpir and was then mobilized in EDL933 by conjugation. Single-crossover integrants of strain EDL933 were selected on M9 agar containing appropriate antibiotics (ampicillin, kanamycin, streptomycin). Selection for double-crossover allele replacement was obtained by *sacB* counter-selection on LB agar plates without NaCl but containing 5% sucrose (Kaniga et al. 1991) and 5-bromo-4-chloro-3-indolylphosphate. To create an EDL933 *phoB* knockout mutant, we performed the allelic exchange using the conjugative donor strain χ7213 (Kang et al. 2002). PCR fragments; upstream (768-bp) and downstream (453-pb) of *phoB* gene were amplified from strain EDL933 using respectively the primers set *Asc*I-PhoB-F/phoB-H1P1-R (5′ggcgcgccgtggcgatgatgggcagagg3′/5′ggtcgacggatccccggaatgatttgccctgttgtaataa3′) and phoB-H2P2-F/*Sac*I-PhoB-R (5′cgaagcagctccagcctacaagccgcgagcagctgttaaa3′/5′gagctcgcggggtcatactgcgatcc3′) and then ligated, using overlap PCR, to the kanamycin resistance cassette from pKD13 plasmid (Datsenko and Wanner 2000) amplified using phoB-H1P1-F/phoB-H2P2-R primers (5′ttattacaacagggcaaatcattccggggatccgtcgacc3′/5′tttaacagctgctcgcggcttgtaggctggagctgcttcg3′). The resulting Δ*phoB*::km construct was digested with *Asc*I and *Sac*I, and then inserted into the conjugative plasmid pMEG-375 cut with the same enzymes. The resulting construct was transferred to strain χ7213, which is a Δ*asd* auxotroph for diamino pimelic acid (Dap) and was then mobilized in EDL933 by conjugation. Double-crossover integrants of strain EDL933 were selected on modified LB agar-containing kanamycin but without Dap. The selected mutant EDL933 derivatives were confirmed to contain a deletion in the *pst* operon or *phoB* gene, respectively, as determined by PCR amplification and sequencing (Eurofins MWG Operon). They were tested for alkaline phosphatase activity that measures the activity of Pho regulon as described previously (Brickman and Beckwith 1975). Antibiotics or supplements were used at the following final concentrations, when required: ampicillin 50 μg mL^−1^, kanamycin 50 μg mL^−1^, streptomycin 100 μg mL^−1^, Dap 12 μg mL^−1^, and 5-bromo-4-chloro-3-indolylphosphate 40 μg mL^−1^.

### Amoebae–bacteria association experiments

Static monocultures and cocultures of amoebae (10^5^ cells mL^−1^) and *E. coli* (10^6^ CFUs mL^−1^) were maintained without agitation at 30°C in PYG medium 1:5 diluted in PBS (Douesnard-Malo and Daigle 2011). Samples (1.2 mL) were taken at days 1, 5, 9, 14, and 21. Total bacteria were estimated by counting the number of colony-forming units (CFUs), with the limit of detection set at 10 CFUs. *A. castellanii* densities were estimated from the same samples by counting the number of amoebae stained with 0.4% trypan blue for a few minutes with a hemocytometer under an inverted microscope. To determine if amoebae provide any bacterial protection during the association experiment, a part of the samples taken at days 9, 14, and 21 were also treated with gentamicin (2 mg mL^−1^) for 2 h prior to amoebae lyses with 0.5% SDS, and then monitored for bacteria by plating followed by CFU counts the next day.

### Estimation of viable but nonculturable bacteria

Bacteria in the viable but nonculturable (VBNC) state are defined as those that fail to grow on routine bacteriological media, but are alive and demonstrate very low levels of metabolic activity (Oliver 2005). The numbers of viable cells were determined using a LIVE/DEAD BacLight Bacterium Viability kit (Molecular Probes Inc., Eugene, Oregon) according to the manufacturer's instructions and as previously described (Yanming Liu et al. 2009). The counts of viable cells (green only) were made with two technical replicates of diluted samples using multispot fluorescence microscopy slides. The VBNC number was calculated by subtracting the cultured number (i.e., CFU) from plate counts from the total number of viable bacteria as determined by fluorescence.

### Transmission electron microscopy

The interactions between the two microorganisms were observed by transmission electron microscopy (TEM). Samples (6 mL) of coculture and each monoculture were centrifuged for 10 min at 800 × *g* and were then washed in PBS. The pellets thus obtained were fixed for 1 h in 2% glutaraldehyde and 0.3% osmium tetroxide prepared in 0.1 mol L^−1^ sodium cacodylate buffer pH 7.3. The samples were centrifuged, dehydrated, and embedded in Epoxy resin prior to being cut into ultrathin section then stained with uranyl acetate and lead citrate. The stained sections were examined with a JEOL, JEM-1230 TEM (Tokyo, Japan) operated at 80 kV.

### Determination of phosphorus level

To evaluate the effect of phosphate availability on amoeba when monocultured and cocultured with *E. coli*, we measured the total phosphorus and the total Pi remaining in the media, at days 14 and 21 of culture. To do this, we used four replicates of the 30-mL amoeba monocultures or cocultures with either EDL933WT or Δ*pst* strains. The supernatants were filtered, adjusted to pH 2.0 then pooled to obtain 100 mL minimal volume required for phosphate measurement by a semiautomated colorimetry method. This was done at the Centre d'expertise en analyse environnementale du Québec (Laval, Canada) using the standard molybdate-reactive P method (AWWA/APHA/WEF 2012).

### Statistical analysis

Statistics were done using GraphPad-Prism 5.0 Software (GraphPad-Prism Inc., San Diego, California). All experiments were conducted in triplicate. *Standard deviation* of the means was calculated from the data of samples of each triplicate time point. A repeated-measures two-way ANOVA and a Bonferroni posttest were used to determine at which time point growth of protozoa differed significantly between absence and presence of different bacterial strains and also to compare bacterial CFUs in the bacteria–amoeba cocultures**.**

## Results

### The presence of *A. castellanii* prolongs EHEC cultivability

**Figure 1:**
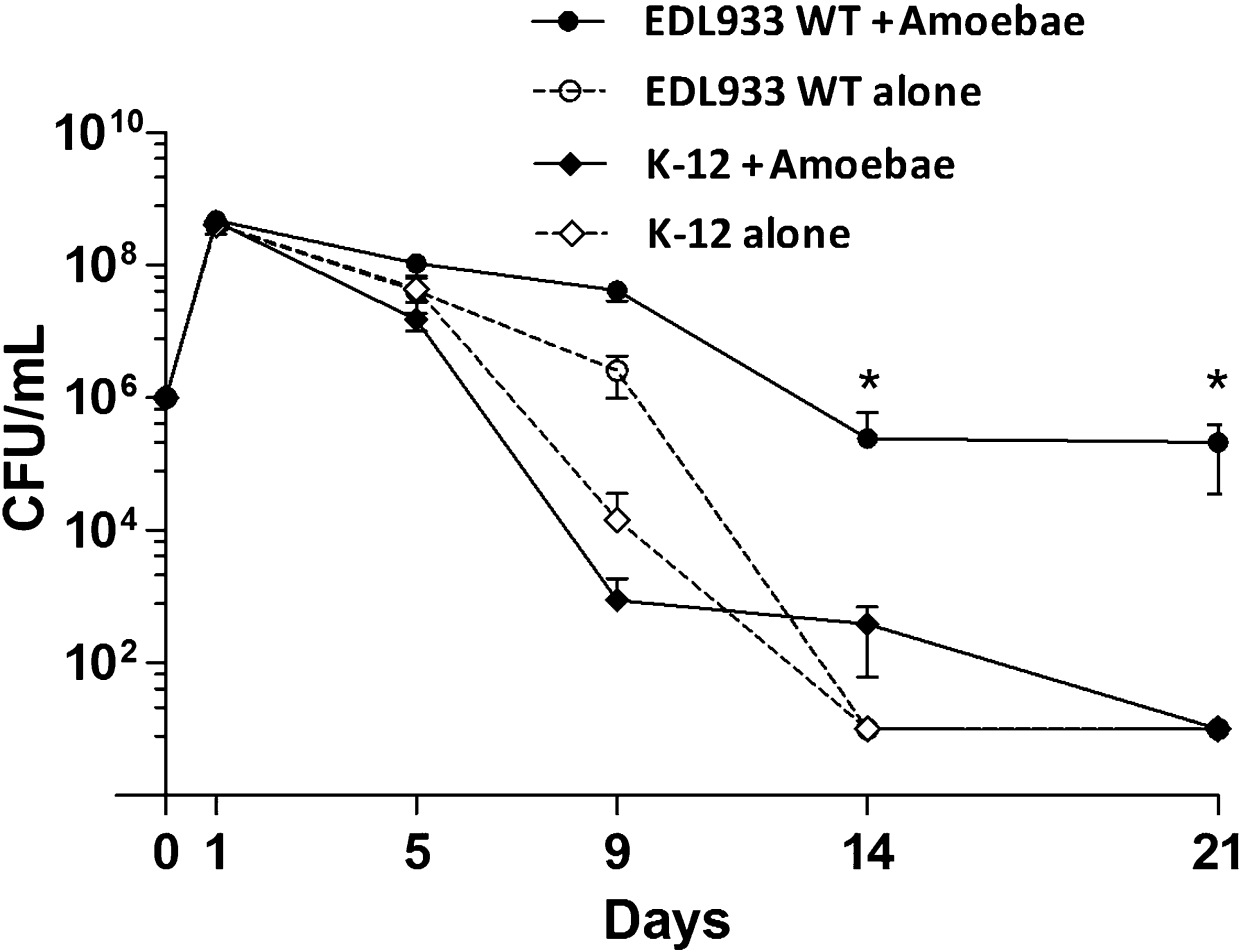
Prolonged cultivability of EDL933 in association with amoebae. *Escherichia coli* EDL933WT (circle symbol) and *E. coli* laboratory strain K-12 HB101 (diamond symbol) were incubated with (solid lines) or without (dotted lines) *Acanthamoeba castellanii* for 3 weeks. The cultivability of EDL933WT maintained and prolonged to days 14 and 21 in coculture with amoebae than without (*P < 0.05). Data indicate mean ± SD of three independent experiments.

The interaction of EHEC O157 strain EDL933 with *A. castellanii* amoeba was investigated by a coculture experiment over a 3-week period. It was possible to establish a typical culture profile of bacteria in the presence or absence of amoebae (Fig. 1). The presence of *A. castellanii* prolonged *E. coli* O157 cultivability after 9 days of coculture. Indeed, individual bacteria showed reduced CFU numbers after day 9 and reached the limit of detection at days 14 and 21 (Fig. 1). Moreover, at day 9 of coculture and after gentamicin (Gm) treatment 10^5^ ± 4.7 × 10^4^ CFU mL^−1^ of strain, EDL933 was recovered after the elimination of extracellular bacteria. However, no bacteria were recovered at day 14 or 21 after Gm treatment (data not shown). In contrast, the nonpathogenic *E. coli* K-12 strain HB101 showed a rapid decrease of CFUs after day 9, either with or without amoebae. The HB101 strain, in addition to the *rfb* rough mutation, carries a *recA* mutation that could be involved in its reduced viability in stress conditions.

**Figure 2:**
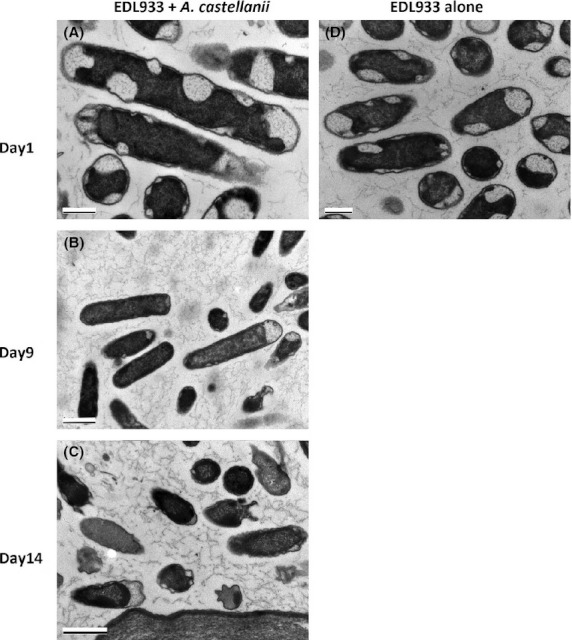
EDL933 morphological change in coculture with amoebae. Electron microscope analysis of EDL933WT in coculture with amoebae at days 1, 9, and 14 (respectively, A, B, and C) and after 1-day monoculture (D). Scale bars: A and D = 1 μm, B and C = 0.5 μm.

Transmission electron microscopy examination of *E. coli* EDL933 monocultured or cocultured with amoebae showed typical and regular rod-shaped bacteria at day 1 (Fig. 2A and D). However, at days 9 and 14 of cocultures, EDL933 exhibits an irregular shape and loss of its initial appearance (Fig. 2B and C). When nonpathogenic *E. coli* K-12 was used in the coincubation experiment, only a few bacteria were still visible at 9 days post incubation (Fig. S1C and D). These remaining bacteria showed an irregular morphology and their cell contents was less uniform compared with those of cells from strain EDL933 after the same incubation period (Fig. S1E).

### The Pho regulon, but not Stx, contributes to EHEC persistence when cocultured with *Acanthamoeba*

**Figure 3:**
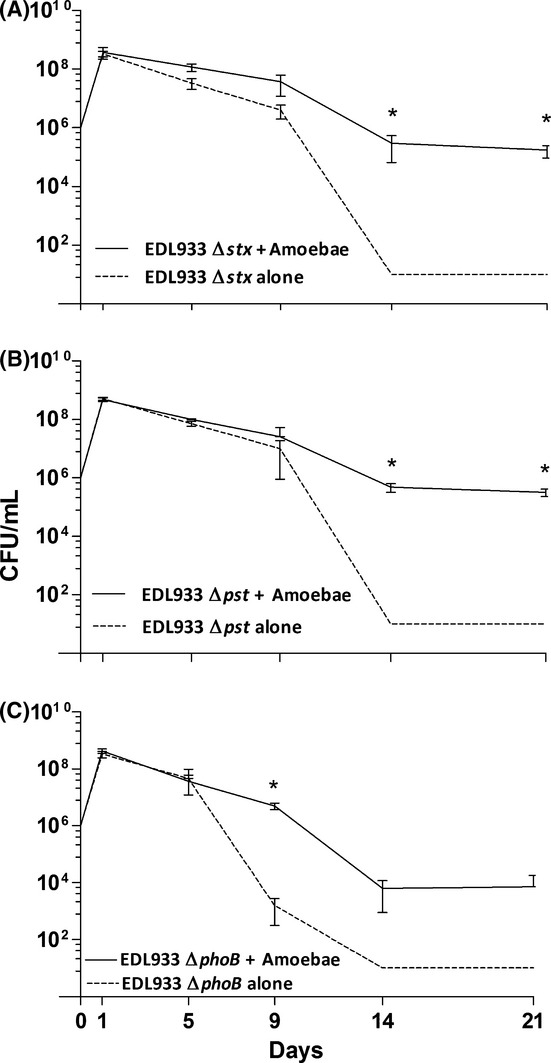
Δ*stx* and Δ*pst* EDL933 mutants are maintained in association with amoebae. *Escherichia coli* EDL933 mutants incubated with (solid lines) or without (dotted lines) *Acanthamoeba castellanii* for 3 weeks. (A. EDL933Δ*stx*; B. EDL933Δ*pst*; and C. EDL933Δ*phoB*). Significant differences were found at days 14 and 21 for Δ*stx* and Δ*pst*. The strain EDL933Δ*phoB* mutant associated with amoebae showed statistical difference only at day 9. (*P < 0.05). Data indicate the mean ± SD of three independent experiments.

To determine the effects of one of the major EHEC virulence factors during its interaction with amoebae, the coculture experiments were repeated using an EHEC strain defective in Stx toxins production (EDL933Δ*stx*). The results were similar to wild-type EDL933 (EDL933WT; compare Figs. 1 and 3A). As with the WT strain, EDL933Δ*stx* significantly persisted in the presence of amoebae compared to when grown as pure culture (Fig. 3A).

To investigate the role of the Pho regulon during interactions with amoebae, we used the Pho constitutively activated strain (EDL933Δ*pst*) and the Pho-inactivated strain EDL933Δ*phoB* (Hsieh and Wanner 2010). Similar to EDL933WT, the CFU of the Δ*pst* mutant was significantly higher after day 9 in the presence of amoebae (Fig. 3B). On the other hand, enumeration of EDL933Δ*phoB* cocultured with amoebae showed a 100-fold CFU reduction compared with WT strain at days 14 and 21. In addition, the EDL933Δ*phoB* monoculture exhibited a decreased cultivability to 10^3^ CFU mL^−1^ at day 9 and then reached the limit of detection at day 14 until the end of the experiment (Fig. 3C).

**Figure 4:**
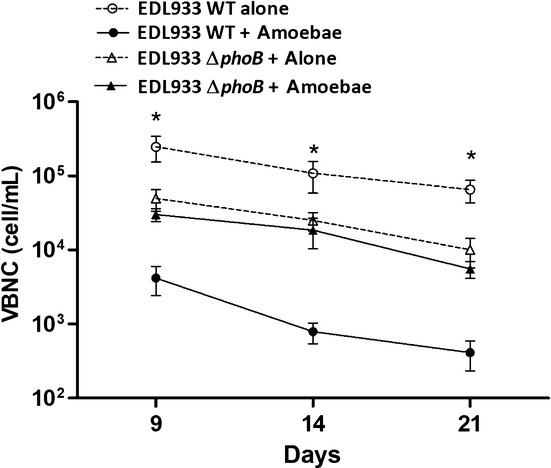
Viable but nonculturable (VBNC) state of *Escherichia coli* EDL933. Calculations of VBNC of EDL933WT (circle symbol) and its mutants EDL933Δ*phoB* (triangle symbol) during 3 weeks in coculture with *Acanthamoeba castellanii* (solid lines) or without (dotted lines). Samples were taken at days 9, 14, and 21. The number of the WT VBNC in monocultures was significantly increased compared with coculture with amoebae (*P < 0.05). In contrast, no similar significant difference was observed for the VBNC number of the Δ*phoB* strain.

Next, we determined the number of viable and dead bacteria at the sampling time points of days 9, 14, and 21 as described in the Materials and Methods. These were monitored, respectively, by green and red fluorescence using the LIVE/DEAD BacLight Kit. These were followed by the estimation of the number of bacteria in a VBNC state. As shown in Figure 4, the absence of amoebae significantly increased the number of EDL933WT cells in a VBNC state compared with the presence of amoebae (*P* < 0.05). However, for the EDL933Δ*phoB* strain, we found no significant VBNC differences between the presence and absence of amoebae. In coculture experiments, even the EDL933Δ*phoB* cultivability defect became observable as of day 9, there was no difference in VBNC when the EDL933Δ*phoB* strain was grown in monoculture or in coculture with amoebae. Thus, inactivation of the Pho regulon resulted in bacterial entrance into a VBNC state, regardless of the presence or absence of amoebae. In addition, there were fewer EDL933WT VBNC cells in coculture with amoebae than EDL933Δ*phoB* VBNC in monoculture or cocultured with amoebae. For the other EHEC mutant strains (Δ*pst,* Δ*stx*), the VBNC results were similar to those obtained for the WT strain (data not shown).

### EHEC reduces the growth rate of *A. castellanii*

**Figure 5:**
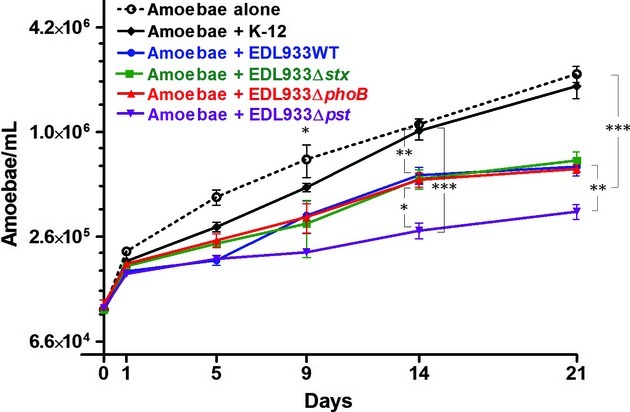
The growth of *Acanthamoeba castellanii* decreases in the presence of EDL933 and mutant derivatives. Amoebae were grown in PYG medium at 30°C for 3 weeks of static monoculture (dotted lines) or cocultured with *Escherichia coli* strains (solid lines). Significant decreases in growth of amoebae in the presence of either EDL933WT or its isogenic mutants (Δ*stx,* Δ*pst*, and Δ*phoB*) compared with amoebae in monoculture or cocultivated with *E. coli* K-12. (***P < 0.001*,* **P < 0.01, *P < 0.05). Results are the mean ± SD of three independent experiments.

Amoebae cell counts were compared between individual cultures and cocultures with *E. coli* strains (Fig. 5). In these in vitro conditions, the presence of the EDL933WT strain and all mutant derivatives (Δ*stx,* Δ*phoB* and Δ*pst*) significantly reduced the growth of the amoebae after 9 days of coculture, and this reduction was more apparent at 14 and 21 days of coculture especially for the Pho constitutive mutant EDL933Δ*pst* (*P* < 0.01/*P* < 0.001; Fig. 5). However, during coculture with the nonpathogenic *E. coli* K-12 strain, no significant differences in amoebae growth or morphology were observed.

**Figure 6:**
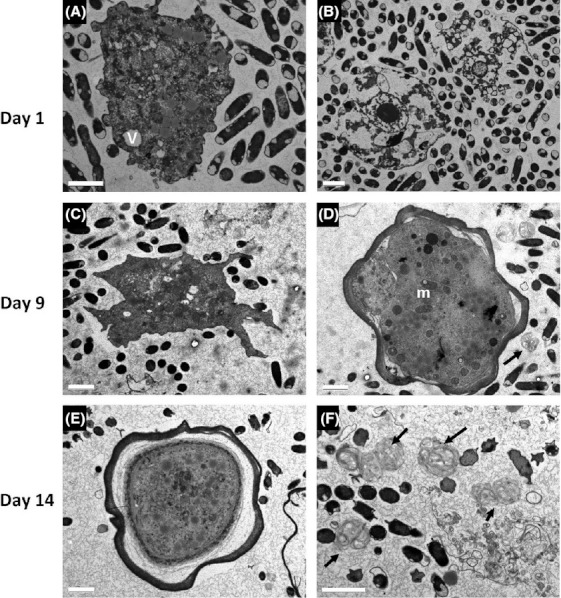
*Acanthamoeba castellanii* morphology in coculture with EDL933. Electron micrographs showing the extracellular localization of EDL933 and the effect on amoebae morphology. *Acanthamoeba castellanii* trophozoites alive (A) and killed (B) in coculture with EDL933 at day 1 and at day 9 (C, D). A mature cyst formed by *A. castellanii* at day 14 (E). Multilamellar bodies secreted by amoebae (black arrow) at day 9 (D) and day 14 (F). M, mitochondria; V, vacuole. Scale bars = 2 μm.

Transmission electron microscopy observations were made to visualize the fate of *A. castellanii* in presence of bacteria (Figs. 6 and S1). Despite the nonsignificant change of the viable amoebae number at day 1 between monoculture and coculture experiments with EDL933, the TEM observations showed that some amoebae cells were damaged by *E. coli* O157 (Fig. 6B). This was concomitant with the highest number of bacteria recovered at day 1 postincubation (Fig. 1). For instance, we were not able to observe any intracellular localization of EDL933 during the 3-week period. However, we observed that most *Acanthamoeba* trophozoites turned into mature cysts at days 9 and 14 (Fig. 6E) while a significant number of multilamellar vesicles were secreted by amoebae (Figs. 6D and F, S1F).

As the Pho regulon is constitutively activated in the *pst* mutant, this mutant is fitted for phosphate competition with the amoebae. The concentration of phosphate remaining in media was determined after 14 and 21 days of culture. The dosages of total phosphate in a pool of four replicates of amoebae monoculture and cocultures were, respectively, 110 and 130 mg mL^−1^. In the same cultures, the concentrations of total Pi were slightly different between absence and presence of either EDL933WT or Δ*pst* (respectively, 102.6, 108.9, 113.4 mg mL^−1^ at day 14 and 103.5, 117, 113.4 mg mL^−1^ at day 21). Thus, the phosphate availability failed to explain the drastic reduction of amoebae in coculture with the EDL933Δ*pst* strain.

## Discussion

This report explores the interaction of EHEC with *A. castellanii*. EHEC was able to survive for 21 days in coculture with *A. castellanii* while an *E. coli* K-12 strain rapidly declined during coculture and was not detected after 21 days. Moreover, the growth of *A. castellanii* was restricted in the presence of EHEC. In addition, the amoebae were damaged by EHEC. However, Shiga toxins appeared to be uninvolved in this long-term interaction as the amoebae growth with *stx* mutant declined similarly to that with the wild type. Other studies have shown that the presence of Stx-encoding prophage augmented the fitness of *E. coli* in coculture with *Tetrahymena* (Steinberg and Levin 2007) and that Stx-positive *E. coli* killed the amoebae (Lainhart et al. 2009). In contrast, another report indicated that Shiga toxin lysogenic phage conferred no advantages in *E. coli* interaction with ruminant ciliates (Burow et al. 2005). Thus, the contribution of Shiga toxins to bacterial survival when facing a protozoan seems to be variable depending on the conditions of the challenge and the protozoan used in the coculture assay.

The decrease of amoebae in coculture with EHEC can be due to the toxicity of EHEC, its capacity to resist digestion, and/or a better capacity of the bacterial strain to compete for nutrients during culture. It was shown by Ravva and coauthors that protozoa in dairy lagoon wastewater were capable of consuming but not eliminating EHEC by retaining them in food vacuoles (Ravva et al. 2010). Moreover, this group showed that the protozoan *Vorticella microstoma* ingested EHEC without digesting them. Protozoan grazing is a major trophic pathway whereby the biomass reenters the food web (Khan 2009). Nonetheless, protozoa do not digest all bacteria. Those known to evade digestion will result in their increase in the environment (Huws et al. 2008). In the case of EHEC, the association with the amoebae allows the bacteria to persist**.**

In prolonged coculture, the EDL933Δ*pst* strain reduced the growth of *A. castellanii* even more than EHEC WT while no significant differences were found for phosphate concentrations between the presence of EHEC WT and Δ*pst* in the prolonged coculture. Thus, the mechanism underling the adverse effect on amoebae growth remains unknown. Similarly, *Pseudomonas aeruginosa* was also found to suppress the growth of *A. castellanii* (Wang and Ahearn 1997). In this condition, it has been suggested that dependent on functional RhlR/LasR quorum-sensing systems, *P. aeruginosa* formed microcolonies and biofilms that exhibited acute cytotoxicity against the protozoa (Matz et al. 2004). In the case of EHEC especially EDL933Δ*pst* strain, further investigations are needed to examine if such phenomenon is involved in the interaction with the amoebae.

Interestingly, some studies have shown a relationship between the Pho regulon and biofilm formation (Lamarche et al. 2008). It is also possible that the *pst* mutant may be more adapted to compete with amoebae in an environment that are limited in nutrients (Lamarche et al. 2008). This is supported by the observed increase of expression of some Pho regulon genes (*phoA*, *pstA,* and *pstB*) of EDL933 when facing *A. castellanii* (Carruthers et al. 2010). The Pst system is involved in phosphate acquisition as well as the molecular mechanisms that lead to turning off the Pho regulon. Not only Pho regulon is a global regulatory circuit involved in bacterial phosphate management but it could also alter other cellular responses and virulence traits that could affect the bacterial survival in association with amoebae (Lamarche et al. 2008; Hsieh and Wanner 2010). However, it was observed that the virulence of the *pst* mutant from other *E. coli* pathotypes such as extraintestinal pathogenic *E. coli* was attenuated (Bertrand et al. 2010; Crepin et al. 2011) demonstrating the great complexity related to the study of bacteria–protozoa interactions.

In adverse environmental conditions, the bacterial cells can lose cultivability but remain viable. In these situations, bacteria reduce general metabolic activity and enter into a VBNC state (Oliver 2005). Interestingly, the presence of amoebae reduces the number of EHEC cells in the VBNC state, suggesting that coculture with amoebae enhances EHEC fitness and persistence during prolonged periods. This could result from amoebae metabolites secretion or nutrients released from amoebae dead cells that could be useful for the surviving EHEC. Furthermore, our results suggest that increased presence of metabolically active and culturable bacteria in association with amoebae requires PhoB activity, because the EDL933Δ*phoB* strain exhibits similarly high number of VBNC whether cultures in the presence or absence of amoeba. Similarly, it has been demonstrated that *Acanthamoeba* promotes survival of *Legionella pneumophila* after disinfection and resuscitate cells from VBNC (Garcia et al. 2007).

Our study shows that EHEC association with amoebae allows bacteria to persist, and for the first time, we observed multilamellar vesicles secreted by *Acanthamoeba* in coculture with *E. coli*. These multilamellar structures resemble those produced by *Dictyostelium discoideum*, another predacious amoeba, when incubated with comestible bacteria. These multilamellar vesicles are thought to be the accumulation of undigested products (Gezelius 1959; Hohl 1965). For *A. castellanii*, these structures were observed when the protozoan was incubated in the presence of *L. pneumophila*. In this particular case, bacteria were found packaged in these multilamellar vesicles (Berk et al. 1998; Bouyer et al. 2007). The packaging of *L. pneumophila* in multilamellar vesicles is known to protect the bacteria from harsh conditions and increase their viability (Rowbotham 1980; Berk et al. 1998; Bouyer et al. 2007). In the case of EHEC, no bacteria were seen in these structures by TEM. Such structures could contribute to a protective microenvironment and/or supplemental nutrients allowing a survival advantage to EHEC during prolonged growth conditions. All together, our results support the probable association between EHEC and amoebae in their natural ecosystem such as water, soil, feces, and cattle. This is in line with the previous work showing the role of *Acanthamoebae* species as host for *Vibrio cholerae* and *Vibrio mimicus* found in the aquatic environments of cholera endemic areas (Abd et al. 2010; Sandstrom et al. 2010). Indeed, *V. cholera* and EHEC share similarities being water-borne and causing diarrheal disease and generally believed to be extracellular bacteria. However, in relation to *A. castellanii*, *V. cholerae* behaved as a facultative intracellular bacterium and, under the experimental conditions used, apparently established a symbiotic relationship with the amoebae (Greub and Raoult 2004). In our study, although the interaction with the *A. castellanii* is beneficial to EHEC growth, we do not observe a similar behavior by electron microscopy.

This study describes one possible lifestyle of the EHEC and may contribute to understanding its ecology that may lead to potential strategies to fight against their transmission to humans and/or the recontamination of ruminants. We demonstrated that a free-living protozoan *A. castellanii* contributes to the long-term persistence of EHEC. Considering the small number of EHEC required for an infectious dose, the role of protozoa in food and water contamination warrants greater focus in prevention research.

## Supporting Information

**Figure S1.** *Acanthamoeba castellanii* alone and in coculture with *Escherichia coli* K-12 HB101. Electron micrographs showing the two forms of *A. castellanii* in monoculture, the metabolic active trophozoite (A) and the dormant cyst (B) and also in coculture with *E. coli* K-12 HB101 (C and D). Note that few *E. coli* K-12 HB101 cells are still visible at day 9 of coculture (E) and multilamellar vesicles are also secreted in this condition (F). Scale bars: A–D = 2 μm, E = 1 μm, F = 0.5 μm.
