# Supplementary figures and images for: Survival of enterohemorrhagic Escherichia coli in the presence of Acanthamoeba castellanii and its dependence on Pho regulon
Source: Microbiologyopen. 2012 Oct 30;1(4):427–37. doi: 10.1002/mbo3.40 (PMC3535388; doi:10.1002/mbo3.40)

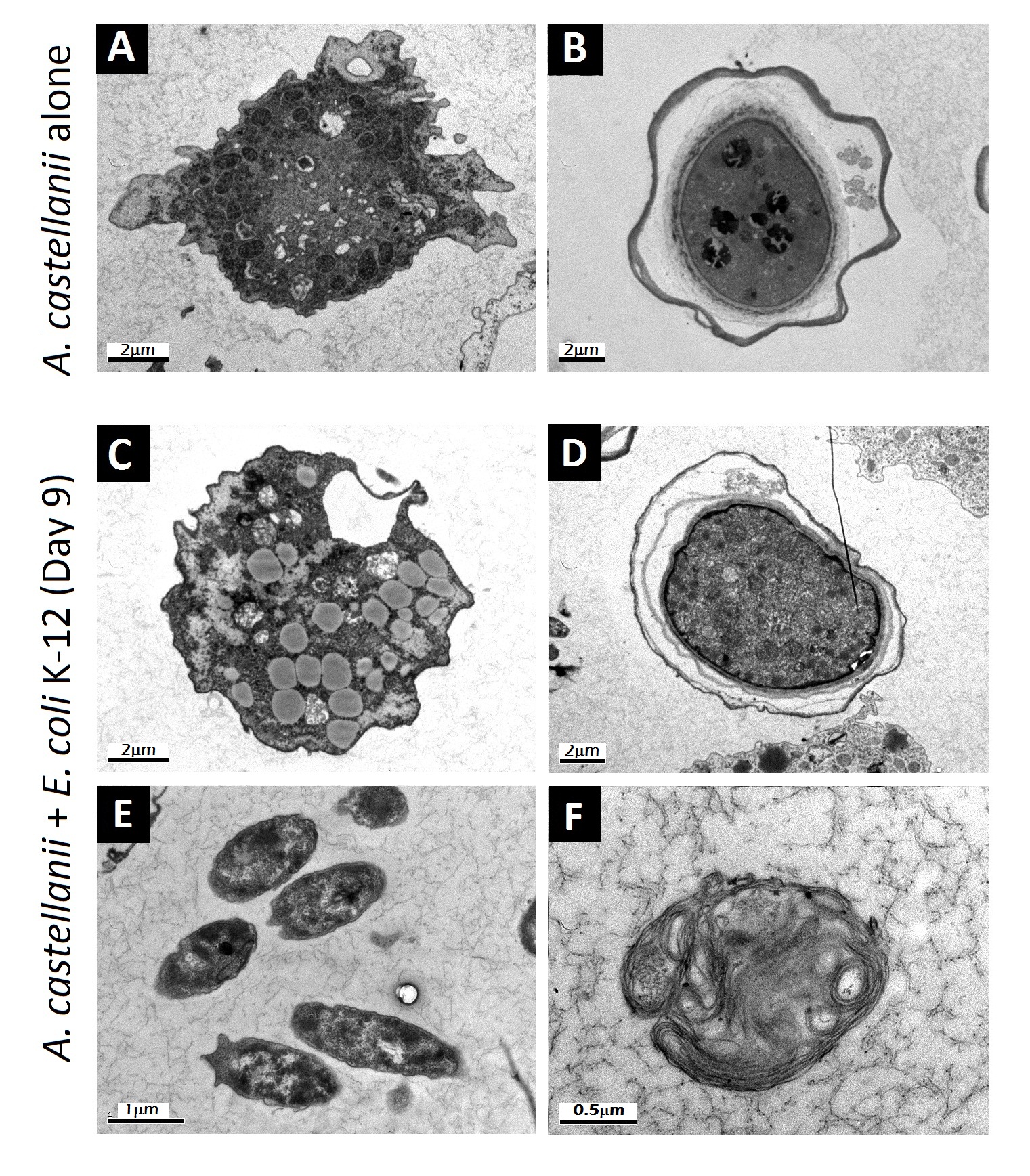

Supplement: Supplementary file 1 [file mbo30001-0427-SD1.jpg]
