# Supplementary material for: Survival of enterohemorrhagic Escherichia coli in the presence of Acanthamoeba castellanii and its dependence on Pho regulon
Source: Microbiologyopen. 2012 Oct 30;1(4):427–37. doi: 10.1002/mbo3.40 (PMC3535388; doi:10.1002/mbo3.40)
Supplement: Supplementary file 2 [file mbo30001-0427-SD2.docx]

**Supporting information**


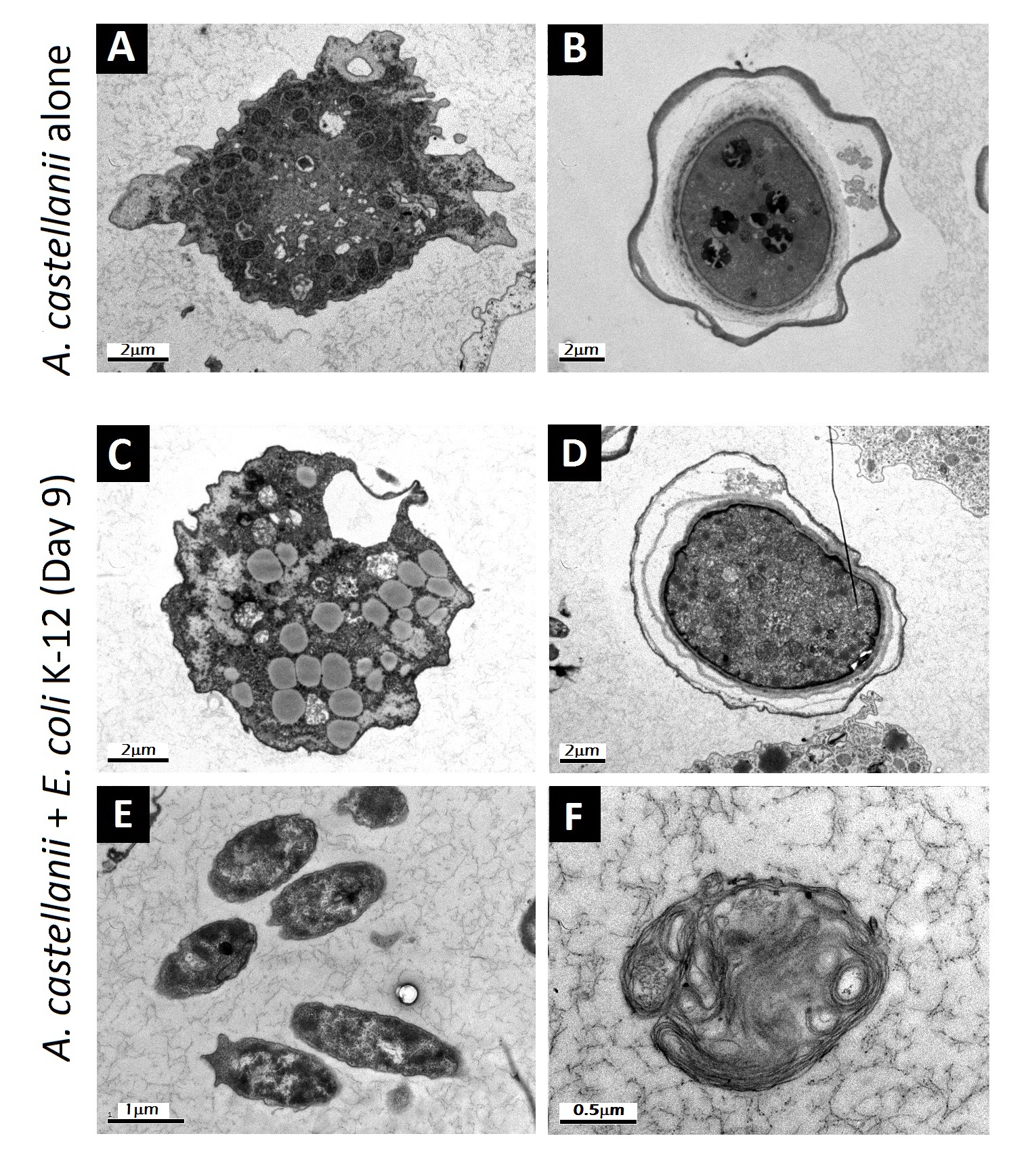


**Figure S1. *A. castellanii* alone and in co-culture with *E. coli* K-12 HB101.** Electron micrographs showing the 2 forms of *A. castellanii* in monoculture, the metabolic active trophozoite (A) and the dormant cyst (B) and also in co-culture with *E. coli* K-12 HB101 (C and D). Note that few *E. coli* K-12 HB101 cells are still visible at day 9 of co-culture (E) and multilamellar vesicles are also secreted in this condition (F). Scale bars: A to D= 2 µm, E= 1 µm, F= 0.5 µm.
